# Soil health to human health: impacts of soil contaminants on cardio-neurological health and clinical outcomes: a narrative review

**DOI:** 10.3389/fcvm.2026.1647648

**Published:** 2026-05-07

**Authors:** Ayoola Awosika, Mayowa Jeremiah Adeniyi

**Affiliations:** 1 Department of Family and Community Medicine, University of Illinois College of Medicine Peoria, Bloomington, IL, United States; 2 Department of Physiology, University of Rwanda, Kigali, Rwanda

**Keywords:** cardiometabolic outcomes, cerebrovascular disease, fertilizers, nutrition security, One Health, pesticides, soil health, soil pollutants

## Abstract

The nexus between soil health and human health represents a critical yet underexplored dimension of cardio-neurological disease research. Soil constitutes the primary ecological substrate determining food quality, nutrient density, and ultimately nutrition security. However, progressive soil degradation and contamination by heavy metals, pesticide residues, persistent organic compounds, and microplastics within agricultural systems and the human food chain have reshaped disease risk profiles. Despite extensive investigation of air and water pollution, the intersection between soil contaminants and cardiovascular and neurological outcomes remains comparatively undercharacterized, revealing a significant knowledge gap between environmental and clinical medicine. Mechanistically, chronic ingestion of soil-derived toxicants promotes oxidative stress, mitochondrial dysfunction, endothelial injury, and neuroinflammation, while disrupting calcium signaling, lipid metabolism, and vascular autoregulation. Fertilizers, animal waste, pesticides, and organic pollutants function as endocrine-disrupting chemicals, activating the aryl hydrocarbon receptor (AhR) to mimic or impair normal endocrine and ligand signaling. In parallel, depletion of essential micronutrients from degraded soils reduces antioxidant capacity and impairs cardiometabolic and neuronal resilience. This dual burden of toxic exposure and diminished nutritional protection provides a plausible pathophysiologic framework linking contaminated soils to hypertension, atherosclerosis, ischemic stroke, cognitive impairment, and neurodegenerative processes, thereby influencing both acute and long-term clinical outcomes. From a public health perspective, compromised soil quality undermines nutrition security even where caloric supply is sufficient, subtly amplifying chronic disease risk at the population level. Hence, the integrative paradigm of healthy soil, healthy food, healthy people, and healthy planet highlights the necessity of transdisciplinary research, improved soil stewardship, and preventive strategies that recognize soil ecosystems as upstream determinants of human cardio-neurological health. Bridging the soil-to-heart-and-brain continuum offers transformative potential for precision prevention and sustainable global health, enabling earlier prevention, more precise dietary guidance, and evidence-based policies.

## 1 Introduction

Soil health constitutes a foundational yet frequently overlooked determinant of human health, operating at the interface of environmental integrity, agricultural productivity, and chronic disease epidemiology. Globally, an estimated one-third of soils are moderately to severely degraded due to industrialization, mining, urbanization, intensive agriculture, and climate-related stressors, with many regions demonstrating measurable accumulation of soil contaminants such as heavy metals, pesticide residues, persistent organic pollutants, and emerging contaminants including microplastics (1). These soil pollutants enter the human food chain through plant uptake, bioaccumulation, and trophic transfer, influencing food quality, nutrient density, and long-term nutrition security. While the epidemiologic burden of cardiovascular disease and neurological disorders continues to rise worldwide, environmental attribution has focused predominantly on air and water pollution, leaving soil pollutant exposure comparatively undercharacterized as a contributor to cardio-neurological morbidity and mortality (2, 3).

From a pathophysiologic perspective, soil-derived toxicants exert multisystem effects that converge on vascular and neural tissues. Chronic low-dose exposure to heavy metals and organic pollutants induces oxidative stress, mitochondrial dysfunction, endothelial injury, and systemic inflammation—central mechanisms in atherosclerosis, hypertension, and thrombotic disease (4, 5). Disruption of nitric oxide (NO) signaling, autonomic dysregulation, and epigenetic modification further accelerate vascular remodeling, while neurotoxic contaminants cross the blood–brain barrier, promoting microglial activation, synaptic injury, and protein misfolding pathways implicated in cognitive decline and neurodegeneration (6, 7). Organic contaminants can also exert cerebrovascular toxicity through activation of the aryl hydrocarbon receptor, which modulates vascular gene expression, disrupting lipid metabolism and enhancing cytokine production (8). These mechanisms compromise blood–brain barrier integrity and elevate the risk of cerebral ischemia, microvascular damage, and neuroinflammation. The pathophysiologic impacts of these contaminants may also amplify traditional risk factors such as smoking and obesity, adding complexity to cerebrovascular risk profiles (9). Importantly, soil degradation also diminishes essential micronutrients such as magnesium, selenium, and zinc in crops, weakening antioxidant defenses and cardiometabolic resilience, thereby creating a dual burden of toxic exposure and reduced nutritional protection (10).

Many population-based studies have also demonstrated that prolonged exposure to such contaminants is associated with modifiable risk factors including hypertension, insulin resistance, and hyperlipidemia, which are pathophysiologic precursors to many cardio-neurological pathologies (7). Nonetheless, causal relationships remain underexplored in prospective studies, signaling a major gap in longitudinal data directly linking soil exposure to adverse cardiometabolic and cerebrovascular outcomes. Despite growing recognition of environmental determinants of cardiometabolic and neurological disease, substantial knowledge gaps remain regarding the specific contribution of soil contaminants to cardio-neurological outcomes. Most epidemiologic and mechanistic studies have focused on air and water pollution, while soil-mediated exposures—particularly those affecting nutrient density, chronic low-dose toxicant ingestion, and long-term vascular and neuroinflammatory pathways—remain insufficiently characterized in clinical and population-based research. Furthermore, few studies integrate soil chemistry, nutritional epidemiology, and clinical risk stratification to clarify exposure–disease relationships across the life course.

The aim of this review is to synthesize current evidence on the epidemiology, pathophysiologic mechanisms, and clinical implications linking soil health to cardiovascular and neurological outcomes, while highlighting translational and public health relevance. By examining how soil pollutant exposure and soil-driven alterations in nutrition security influence disease risk and clinical trajectories, this work seeks to advance a multidisciplinary framework that informs preventive strategies, research priorities, and policies promoting healthy soil, healthy food, healthy people, and a healthy planet.

## 2 Methodology

This narrative review integrates and critically examines contemporary evidence addressing the influence of soil pollutants on modifiable risk factors associated with cardio-neurological morbidities. A structured search of major electronic databases—including PubMed, Medline, EBSCO, SCOPUS, and Google Scholar—was conducted using combinations of key terms such as “soil health,” “nutrition security,” “cardiometabolic outcomes,” “cerebrovascular disease,” “organic and inorganic soil pollutants,” and “environmental determinants of health.” Peer-reviewed studies published through 2025 were eligible for inclusion, and additional relevant articles were identified through manual review of reference lists. Priority was given to robust evidence sources—including large-scale population-based cohort studies, disease registries, and meta-analyses—to ensure comprehensive coverage of epidemiologic and mechanistic insights. In keeping with the conventions of narrative reviews, a formal assessment of methodological quality or risk of bias was not performed. Rather than quantitatively pooling results, this review aims to synthesize and interpret existing data to clarify clinically meaningful associations, delineate plausible biological mechanisms, and highlight cardiometabolic and cerebrovascular outcomes linked to environmental exposure to soil-derived pollutants.

## 3 Review

### 3.1 Soil health, contaminant burden, and nutrient density

**Figure 1:**
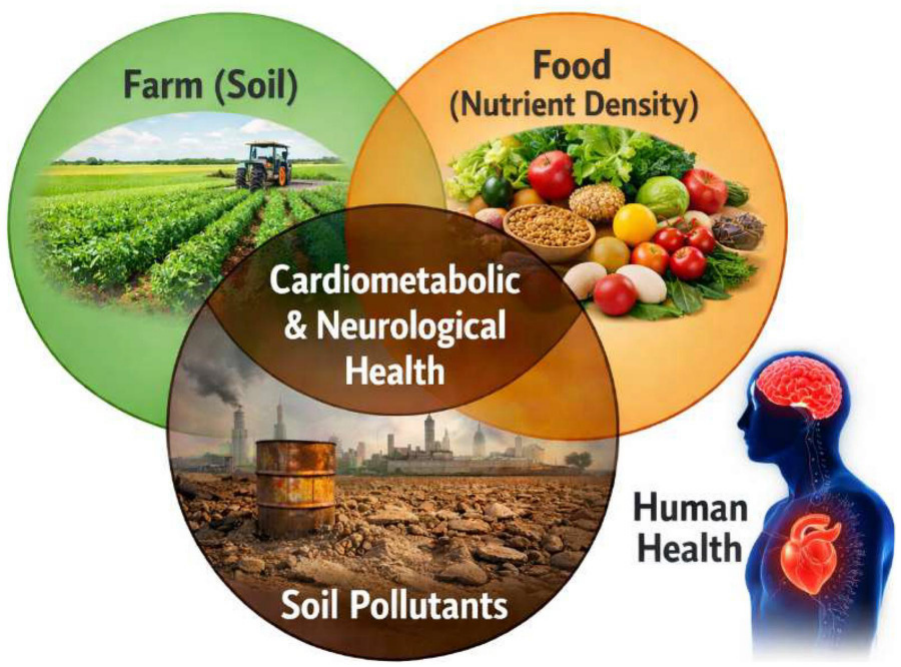
Intersection between soil health, nutrient, and human health.

Soil health is defined not only by its physicochemical structure and microbial diversity but also by its capacity to sustain nutrient cycling and crop micronutrient composition. Accumulation of soil pollutants—including heavy metals (e.g., cadmium, lead, arsenic), persistent organic pollutants, and excess agrochemicals—disrupts rhizosphere microbiota, impairs enzymatic mineralization, and alters cation exchange dynamics, thereby reducing plant uptake of essential micronutrients such as zinc, iron, selenium, and magnesium (11, 12). Cadmium, for example, competes with zinc transporters in plant roots, while arsenic interferes with phosphate assimilation, directly diminishing nutrient density in edible crops (13). Epidemiologically, global soil degradation affects approximately one-third of arable land, with measurable micronutrient decline documented in staple crops across Asia and sub-Saharan Africa (14). Such reductions in nutrient density compromise dietary quality even in food-sufficient regions, linking soil contamination to subclinical deficiencies that predispose to cardiometabolic and neurocognitive vulnerability, as shown in Figure 1.

### 3.2 Soil pollutants, nutrition security, and population-level risk

Beyond crop yield, soil contamination exerts broader implications for nutrition security by simultaneously lowering micronutrient availability and introducing toxic exposures into the food chain. Chronic low-dose ingestion of metal-contaminated produce has been associated with increased biomarkers of oxidative stress, endothelial dysfunction, and neuroinflammation in exposed populations (15, 16). Large population-based studies have demonstrated correlations between elevated soil or dietary cadmium and heightened risks of hypertension, chronic kidney disease, and cerebrovascular events, while arsenic exposure has been linked to impaired neurodevelopment and cardiometabolic dysregulation (16, 17). Importantly, regions with intensive industrial or agricultural activity often experience the dual burden of reduced nutrient density and contaminant bioaccumulation, amplifying disparities in environmental health outcomes (18). Addressing soil pollutant exposure is therefore integral not only to ecological sustainability but also to safeguarding long-term nutrition security and preventing chronic disease.

### 3.3 Overview of agro-allied soil contaminants

Prior to the industrial age, agricultural practices relied primarily on the use of simple tools and less toxic natural substances for the cultivation of plants and livestock, improvements of farm produce, and prevention, management, and control of diseases (19, 20). With great advances in science and technology, the development of modern and sophisticated tools, chemicals for pest control, and yield-improving agents has been heralded as a major breakthrough in the 21st century. Consequently, all these innovations have significantly contributed to soil contamination by introducing cardiometabolic and cerebrovascular toxicants such as artificial pesticides, veterinary and animal residues, and organic and inorganic contaminants. Combined with mining, industrial activities, improper waste management, natural disasters, and environmental stressors, these exposures have markedly increased risk factors for cardio-neurological pathologies and heightened the human burden of diseases (21).

### 3.4 Types of agro-allied soil contaminants

#### 3.4.1 Artificial pesticides

Pesticides are chemicals agents—produced naturally by plants or *in vitro*—that can repel or inhibit any intruding organisms (22, 23). Natural pesticides such as capsaicin in chili pepper are produced at physiological levels. In contrast, artificial pesticides—including organochlorines, pyrethroids, organophosphates, and herbicides—are human-made products that exert deleterious impacts on soils, posing health threats to animals, human beings, and the ecosystem at large. Degrendele et al. (24) evaluated the presence of 30 pesticides in 12 soil samples from two selected sites in South Africa using tandem mass spectrometry coupled with high-performance liquid chromatography. Nine pesticides were observed, with chlorpyrifos, carbaryl, and tebuconazole exhibiting peak concentrations of 63.6, 1.10, and 0.212 ng/g, respectively. The daily consumptions through soil and inhalation were estimated to range from 0.126 fg/kg/day for isoproturon to 14.7 ng/kg/day for chlorpyrifos.

Riedo et al. (25) examined how rapid (short-term) or gradual (long-term) introductions of 10 commonly used pesticides affected soil microbial community and diversity using high-throughput sequencing. Gradual pesticide exposure was shown to deplete the relative abundance of prominent fungal taxa, with triazole fungicide and herbicides having the most prominent impacts. In contrast, rapid pesticide introductions caused only temporary derangements. This implies that long-term pesticide use, rather than short-term use, interferes with soil microbial activity, thereby affecting critical microbe-mediated processes such as decomposition and nutrient recycling, which may impair plant nutrient composition and food quality consumed by humans. In Palatine, Germany, Honert et al. (26) evaluated the composition of current-use pesticides and the possible presence of 93 active ingredients in chemicals. Soil samples were collected on a monthly basis for over 1 year across arable, vegetable, and viticulture agricultural sites. Twenty-five current-use pesticide residues were discovered in the soil. The concentrations of the toxic chemicals were found to be roughly unchanged during summer.

#### 3.4.2 Veterinary and animal residues

Veterinary and animal residues are remnant chemicals and agents in animals and animal-derived foods that are deposited in the soil. These include drugs and medicinal products, antimicrobial compounds, hormones, pesticides, pathogens, heavy metals, endocrine-disrupting chemicals, and metabolites (27). They are derived from agents used for growth enhancement, disease treatment, or exposure to toxic chemicals. Indiscriminate utilization of these agents results in their accumulation in animal bodies, posing significant threats to soil ecosystems, human health, and the environment. In Singapore, Sin et al. (28) evaluated the prevalence of veterinary drug residues—a known cause of antimicrobial resistance in human beings—in commonly consumed foods. In particular, the prevalence of macrolides, beta-agonists, fluoroquinolones, and coccidiostats was analyzed through liquid chromatography tandem mass spectrometry. Out of 216 food samples, 9.72% tested positive for veterinary drug residues, with most of the most positive foods being poultry and poultry-derived food products, followed by eggs. The drugs detected included enrofloxacin, ciprofloxacin, lasalocid, clopidol, diclazuril, tilmicosin, and nicarbazin. Clopidol and enrofloxacin were the most prevalent drugs.

Martins et al. (29) assessed the presence of nine synthetic coccidiostat and ionophore residues in samples of poultry muscles from varying production types. A total of 101 turkey and chicken samples were analyzed for nine chemical residues using solid liquid extraction followed by liquid chromatography with tandem mass spectrometry. Approximately 21% of the analyzed samples contained residues of diclazuril, halofuginone, decoquinate, lasalocid, narasin, and salinomycin. In Italy, Roila et al. (30) evaluated the presence of coccidiostats in 353 samples of animal tissues and eggs, along with 262 untreated feed samples collected between years 2012 and 2017 using high-performance liquid chromatography–mass spectrometry. They identified residues of diclazuril, narasin, and violative. Positive feed samples averaged 25%, ranging from 17.2% in 2012 to 28.3% in 2017. Piątkowska et al. (31) evaluated the presence of veterinary residues in lyophilized egg albumen. A total of 85 compounds were analyzed, and enrofloxacin and doxycycline were confirmed in egg albumen samples. Reported concentrations ranged from 5.65 to 596 µg/kg for doxycycline and 0.89 to 134 µg/kg for enrofloxacin.

#### 3.4.3 Organic and inorganic contaminants

Organic and inorganic contaminants include soil-borne toxic chemicals. These chemicals—including chlorinated paraffin, polycyclic aromatic hydrocarbons (PAHs), polychlorinated biphenyls, polybrominated diphenyl ethers (PBDEs), and heavy metals—are known for their carcinogenic, genotoxic, and neurotoxic effects, making them detrimental to human health. They exert their effects through activation of aryl hydrocarbon receptors (15).

##### 3.4.3.1 Organic contaminants

In a study conducted by Moeckel et al. (32), soil samples collected from the Agbogbloshie electronic-waste disposal site in Ghana were analyzed for a range of organic contaminants, including chlorinated paraffins, PAHs, polychlorinated biphenyls (PCBs), and PBDEs. Among these, chlorinated PCBs demonstrated the highest concentrations, exceeding established soil guideline thresholds and posing significant risks to mammalian health and environmental safety.

Similarly, an environmental assessment of soils and sediments from an urbanized river floodplain in Delhi, India, evaluated organochlorine pesticides (OCPs), PAHs, and phenolic compounds in 2018 (33). A total of 54 samples—comprising 27 soil and 27 sediment specimens—were collected and analyzed. Reported concentrations in soil ranged from 639 to 2,112 µg/kg for phenolic compounds, 473 to 1,132 µg/kg for PAHs, and 13 to 41 µg/kg for OCPs. Corresponding sediment concentrations were substantially elevated, ranging from 553 to 20,983 µg/kg for phenolic compounds, 1,685 to 4,010 µg/kg for PAHs, and 4.2 to 47 µg/kg for OCPs. Notably, four-ring PAHs predominated in both soils and sediments, accounting for approximately 51%–76% of total PAHs, while seven recognized carcinogenic PAHs constituted 43%–61% of total PAH burden. Among OCPs, p,p′-DDT was the dominant compound detected in soils, whereas *α*-hexachlorocyclohexane (*α*-HCH) was most abundant in sediment samples. These findings underscore the persistence and toxicological relevance of organic soil pollutants in rapidly urbanizing environments.

Sun et al. (34) investigated the extent of PCB contamination in agricultural soils across the Yangtze River Delta, one of the most industrialized regions of China. Reported PCB concentrations ranged from <0.1 to 130 ng/g (dry weight). Although lower concentrations were not associated with measurable impairment of soil ecological function, the authors noted that chronic exposure may still present potential health risks to nearby populations through environmental and dietary pathways. In a related investigation, Sun et al. (35) quantified several classes of endocrine-disrupting chemicals and persistent organic contaminants in soils from the same region, including OCPs, phthalate esters (PAEs), and PBDEs. The summed concentrations of 15 OCPs, 13 PBDE congeners, and 15 PAEs ranged from 1.0 to 3,520, <1.0 to 382, and 167 to 9,370 ng/g, respectively. These findings highlight the widespread presence of multiple contaminant classes in intensively cultivated and industrialized landscapes, underscoring the potential for cumulative exposure and long-term environmental and human health implications.

##### 3.4.3.2 Inorganic contaminants

Enjavinejad et al. (36) assessed 77 agricultural sites in the southeastern region of Shiraz, Iran, to determine the sources and spatial distribution of heavy metals, including iron (Fe), manganese (Mn), copper (Cu), zinc (Zn), cadmium (Cd), nickel (Ni), and lead (Pb). Risk assessment indices revealed that cadmium and lead contributed the highest total hazard indices, while nickel demonstrated the greatest individual health risk index among the analyzed metals, underscoring its toxicological significance. In South Africa, Kapwata et al. (37) utilized portable spectrophotometry to quantify concentrations of lead, arsenic, cadmium, and mercury in soils from rural Giyani. Notably, 57% of soil samples obtained from highly impacted areas exceeded contamination thresholds for arsenic, indicating substantial environmental burden. Moreover, Shezi et al. (38) investigated heavy-metal contamination in soils within preschool environments located near industrial operations, highlighting potential exposure risks in vulnerable pediatric populations. Collectively, these studies illustrate the widespread distribution of hazardous metals across diverse geographic and socioenvironmental contexts and emphasize their relevance to environmental health and clinical risk considerations.

### 3.5 Soil pollutants as a predictor of cardiometabolic syndrome

Emerging evidence indicates that soil pollutants are increasingly recognized as environmental determinants of obesity and related metabolic disorders. Epidemiologically, populations residing near industrial sites, electronic waste disposal areas, or agricultural regions with intensive pesticide and fertilizer use exhibit higher prevalence of obesity, metabolic syndrome, and type 2 diabetes mellitus (39, 40). For example, longitudinal studies in areas with chronic heavy-metal contamination—including cadmium, lead, and arsenic—have shown positive correlations between urinary or blood levels of these metals and body mass index, waist circumference, and insulin resistance indices (41, 42). Similarly, exposure to persistent organic pollutants (POPs)—such as PCBs, organochlorine pesticides, and PBDEs—through contaminated soil and crop uptake has been associated with adiposity and impaired glucose homeostasis in both adults and children (43).

Mechanistically, soil-derived pollutants influence obesity through disruption of endocrine and metabolic pathways. Heavy metals and POPs act as “obesogens,” interfering with adipogenesis, lipid metabolism, and energy homeostasis. Cadmium and arsenic induce oxidative stress and mitochondrial dysfunction in adipocytes and hepatocytes, promoting adipocyte hypertrophy and ectopic lipid deposition (44). POPs such as PCBs and PBDEs bind to nuclear receptors—including peroxisome proliferator-activated receptors (PPAR*γ*) and liver X receptors—altering adipocyte differentiation and lipid storage (45). In addition, these contaminants trigger epigenetic modifications, including deoxyribonucleic acid (DNA) methylation of genes regulating appetite, energy expenditure, and adipokine signaling, thereby predisposing exposed individuals to long-term metabolic dysregulation (46).

Chronic low-dose soil pollutant exposure may also exacerbate systemic inflammation and insulin resistance via gut microbiome perturbation. Soil contaminants influence microbial composition in crops and subsequently in the human gastrointestinal tract, altering short-chain fatty acid (SCFA) production, intestinal barrier function, and endotoxemia, which collectively contribute to obesity and cardiometabolic dysfunction. These findings underscore the intersection between environmental soil health and human metabolic outcomes, suggesting that soil pollutants can serve as both direct and indirect predictors of obesity, linking ecological degradation to public health risk.

#### 3.5.1 Soil pollutants and hypertension: pathophysiology and molecular mechanisms

Epidemiological studies indicate that populations exposed to contaminated soils—particularly in industrial, urban, and agricultural settings—exhibit significantly higher prevalence of elevated blood pressure and hypertension. For instance, chronic exposure to soil-derived heavy metals such as cadmium, lead, and arsenic has been associated with a 1.3–2.1-fold increased risk of hypertension in cohort studies conducted across Asia, Africa, and North America (16). In Bangladesh, chronic arsenic exposure through contaminated soils and irrigation water was linked to 41% higher odds of systolic hypertension among adults (47). Similarly, analyses from the National Health and Nutrition Examination Survey (NHANES) dataset demonstrated positive dose–response relationships between blood cadmium and lead levels and both systolic and diastolic blood pressure, suggesting bioaccumulation from soil and dietary sources as a significant contributor to population-level hypertension (48).

**Figure 2:**
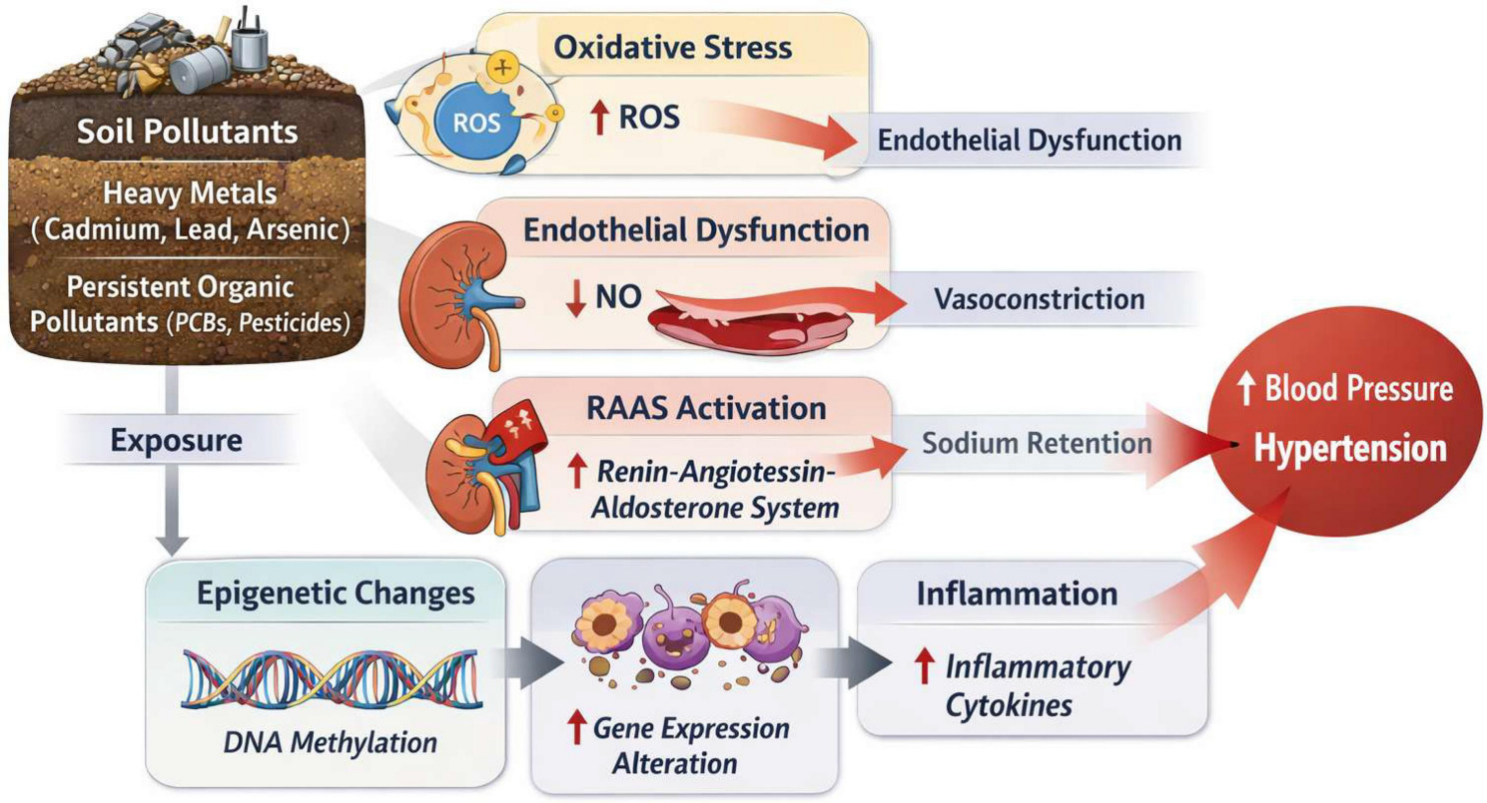
Mechanisms linking soil pollutants to hypertension. ROS, reactive oxygen species; RAAS, renin–angiotensin–aldosterone system; NO, nitric oxide; PCB, polychlorinated biphenyls; DNA, deoxyribonucleic acid.

Pathophysiologically, soil pollutants influence vascular tone and blood pressure regulation through multiple intertwined mechanisms. Heavy metals such as cadmium and lead induce oxidative stress in endothelial cells, promoting reactive oxygen species (ROS) generation, NO scavenging, and impaired vasodilation (49). Arsenic disrupts endothelial function and vascular smooth muscle tone by altering calcium signaling and reducing NO bioavailability, leading to increased systemic vascular resistance (49). Moreover, these metals interfere with renal sodium handling, contributing to salt-sensitive hypertension, while simultaneously activating the renin–angiotensin–aldosterone system (RAAS) and sympathetic nervous system, exacerbating blood pressure elevation (49, 50), as shown in Figure 2.

At the molecular level, soil pollutants act as epigenetic and transcriptional modulators of vascular and renal function. Cadmium and lead exposure has been shown to upregulate nicotinamide adenine dinucleotide phosphate (NADPH) oxidase expression in vascular tissue, augmenting ROS production and oxidative damage, while simultaneously promoting DNA methylation changes in genes involved in endothelial nitric oxide synthase signaling and vascular remodeling (51). Chronic arsenic exposure modulates histone acetylation in renal tubular cells, enhancing angiotensinogen expression and RAAS activation (49, 50). Persistent organic pollutants—such as PCBs and organochlorine pesticides—also contribute to vascular inflammation and dysregulation of lipid and glucose metabolism, indirectly exacerbating hypertension (9, 45).

##### 3.5.1.1 Soil pollutants and hypertension: evidence from studies

In Ecuador, Suarez-Lopez et al. (52) examined the association between pesticide exposure during a low-flower production period and blood pressure in children. The cross-sectional study included 313 children aged 4–9 years residing in agricultural areas, with exposure assessed 63–100 days following harvest. Findings demonstrated a curvilinear relationship between pediatric diastolic blood pressure and postpesticide exposure, with the odds of elevated blood pressure doubling for every 11 days following pesticide application. In addition, acetylcholinesterase activity—an enzyme responsible for acetylcholine degradation—was positively correlated with the postharvest period, suggesting a physiological link between organophosphate exposure and vascular response.

In the United States, Frank et al. (5) investigated the relationship between organophosphate pesticide exposure—measured via dialkyl phosphate metabolites—and blood pressure indices among 916 adults participating in the 2015–2016 NHANES. Elevated levels of diethylphosphate and total diethylphosphate were significantly associated with increased odds of high pulse pressure. Notably, adults in the third quartile of metabolite exposure exhibited more than a 3.5-fold higher likelihood of developing metabolic syndrome compared to those in the first quartile.

Dong et al. (53) analyzed NHANES data from 2007 to 2014 involving 4,260 participants to evaluate associations between non-persistent pesticide exposure and hypertension. Urinary biomarker levels were used to quantify exposure, and weighted logistic regression revealed that para-nitrophenol was positively associated with hypertension. Moreover, cumulative exposure to four pesticide biomarkers demonstrated a significant positive relationship with elevated blood pressure. Expanding on this evidence, Chen et al. (54) examined NHANES data from 1999 to 2014 encompassing 32,309 individuals, reporting that household pesticide exposure was associated with a 30% higher likelihood of hypertension. Collectively, these studies provide convergent epidemiological evidence linking both agricultural and household pesticide exposures to increased blood pressure and hypertension risk.

Exposure to veterinary drug residues—including *β*-adrenergic agonists used therapeutically for conditions such as bronchospasm or premature labor in livestock—represents a potential environmental determinant of hypertension in both healthy and vulnerable populations (55). Of particular concern is the illicit administration of *β*2-adrenergic agonists, such as clenbuterol for growth promotion in cattle—a practice reported in Europe and other regions (56). Consumption of meat or animal products contaminated with clenbuterol has been associated with adverse cardiovascular and cerebrovascular outcomes in humans.

Neary et al. (57) examined the cardiovascular impact of the bovine growth promoter zilpaterol, a *β*2-agonist, in a prospective case–control study involving 80 steers. Results demonstrated that zilpaterol-fed animals exhibited elevated pulmonary diastolic blood pressure, left ventricular hypertrophy, increased interventricular septal thickness, and elevated cardiac troponin I relative to controls, highlighting the cardiotoxic potential of *β*2-agonist residues.

Similarly, bisphenol A (BPA)—a widely prevalent environmental contaminant present in canned beverages and food packaging—has been linked to blood pressure dysregulation. Bae et al. (58) reported that urinary BPA concentrations increased by over 1,600% in adults aged ≥60 years following canned beverage consumption, accompanied by a rise in systolic blood pressure of approximately 4.5 mmHg. Further analyses in a cohort of 521 community-dwelling older adults in Seoul demonstrated a positive association between urinary BPA levels and both elevated blood pressure and impaired heart rate variability (59). Prenatal BPA exposure has also been implicated in pediatric cardiovascular risk. Among 645 children aged 4 years, diastolic blood pressure was positively associated with maternal urinary BPA concentrations measured during midpregnancy (60). Furthermore, Xiong et al. (61) reported that serum BPA levels were significantly higher in 88 patients with dilated cardiomyopathy compared to age- and sex-matched controls, underscoring BPA's potential contribution to structural cardiac abnormalities that predispose to cerebrovascular events.

Chen et al. (62) investigated the relationship between chronic inorganic arsenic exposure and hypertension among 898 residents (382 men and 516 women) living in an arseniasis-hyperendemic village. After adjustment for age and sex, the prevalence of hypertension was approximately 1.5 times higher in residents of the hyperendemic area compared with those in non-endemic communities, suggesting a strong environmental contribution to blood pressure elevation.

In Argentina, Ameer et al. (63) evaluated 225 women with a median urinary arsenic concentration of 200 µg/L to determine associations with blood pressure and early cardiovascular risk markers. Interestingly, arsenic levels demonstrated an inverse relationship with diastolic blood pressure, underscoring potential heterogeneity in exposure–response patterns across populations. In contrast, findings from Bangladesh by Khatun et al. (64)—involving 828 individuals from regions with varying arsenic exposure—revealed a clear dose–response relationship: Increasing arsenic concentrations in drinking water, hair, and nails were associated with progressive elevations in both systolic and diastolic blood pressure.

Similarly, Kaufman et al. (65) analyzed data from 1,910 U.S. participants and reported modest cross-sectional associations between arsenic biomarkers and both systolic and diastolic blood pressure, as well as hypertension prevalence. Wang et al. (66) further demonstrated that among 233 individuals with arsenicosis compared with 84 unexposed controls, arsenic exposure was significantly associated with higher rates of hypertension, widened pulse pressure, and increased systolic blood pressure. Collectively, these studies provide convergent epidemiological evidence linking chronic arsenic exposure to altered blood pressure regulation and heightened hypertension risk, although variations in direction and magnitude of association highlight the complexity of arsenic's vascular effects.

#### 3.5.2 Soil pollutants and hyperlipidemia: pathophysiology and molecular mechanisms

Analyses of large population datasets—including biomonitoring cohorts in Asia, Europe, and North America—have demonstrated significant associations between blood cadmium, lead, and arsenic levels and elevated total cholesterol, low-density lipoprotein (LDL), and triglycerides, independent of traditional risk factors (67). For example, cross-sectional analyses of U.S. population data have reported that individuals in the highest quartile of cadmium exposure had significantly higher odds of dyslipidemia compared with those in the lowest quartile (67). Similarly, studies from arsenic-endemic regions in South Asia have documented altered lipid profiles characterized by elevated triglycerides and reduced high-density lipoprotein (HDL) among chronically exposed populations (68).

**Figure 3:**
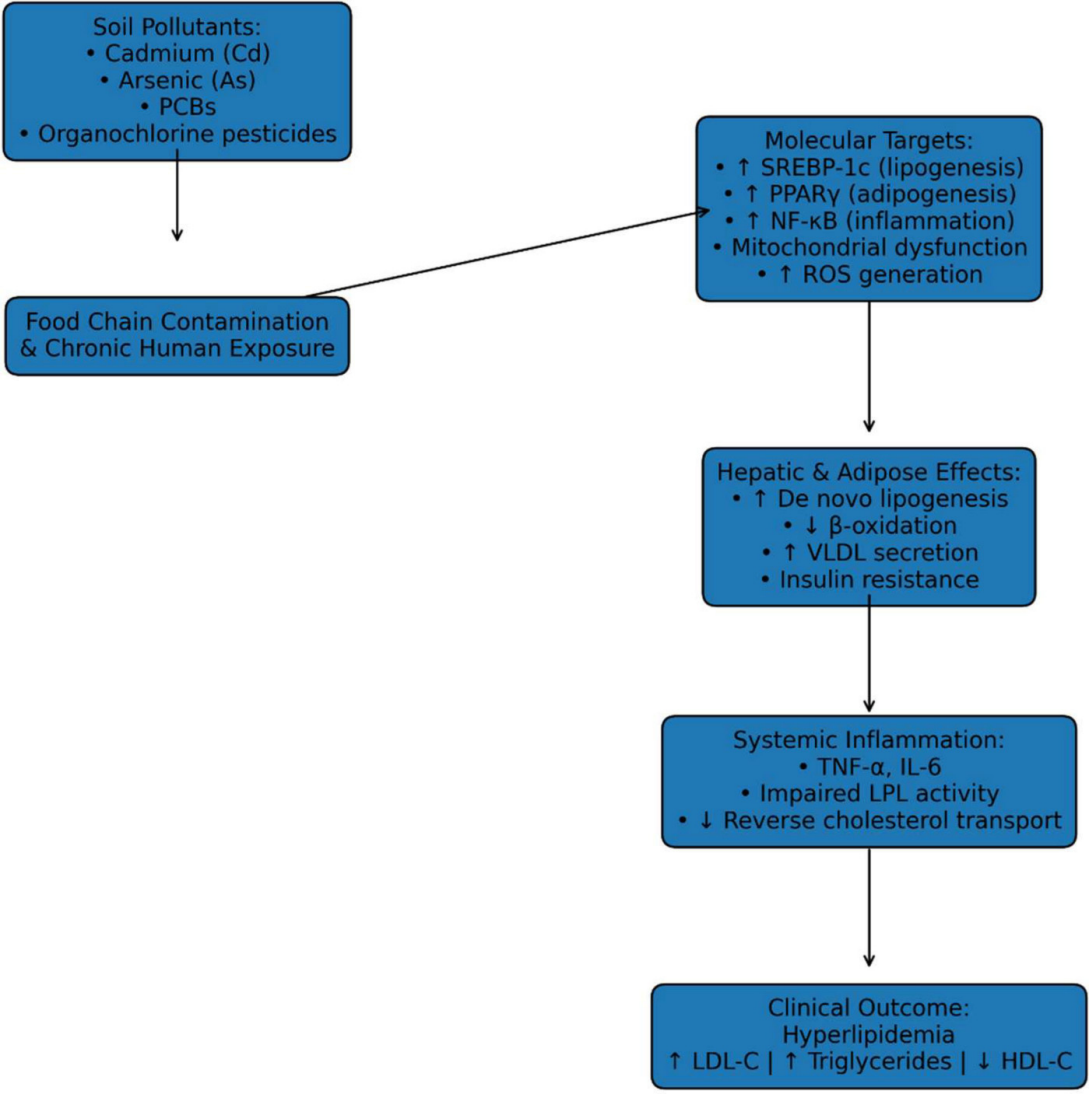
Mechanistic pathways linking soil pollutants to hyperlipidemia. PCB, polychlorinated biphenyls; SREBP, sterol regulatory element-binding proteins; PPAR, peroxisome proliferator-activated receptors; TNF, tumor necrosis factor-α; IL, interleukin-6; ROS, reactive oxygen species; VLDL, very-low-density lipoprotein; LDL-C, low-density lipoprotein C; HDL-C, high-density lipoprotein C.

Heavy metals and organic pollutants disrupt hepatic lipid metabolism, the central regulator of cholesterol synthesis, lipoprotein assembly, and fatty acid oxidation. Cadmium and lead have been shown to impair mitochondrial *β*-oxidation and increase ROS generation in hepatocytes, leading to lipid peroxidation and increased very-low-density lipoprotein (VLDL) secretion (69), as shown in Figure 3. Arsenic interferes with insulin signaling and promotes hepatic steatosis, thereby enhancing triglyceride synthesis and reducing HDL formation (68). In addition, persistent organic pollutants such as PCBs and organochlorine pesticides accumulate in adipose tissue and liver, where they alter transcriptional regulation of lipid metabolism. Experimental and human observational studies demonstrate that POPs activate sterol regulatory element-binding proteins (SREBPs) and peroxisome proliferator-activated receptors (PPARs), key transcription factors governing lipogenesis and adipocyte differentiation, thereby promoting dyslipidemia and ectopic fat deposition (70).

At the molecular level, chronic exposure to soil pollutants induces systemic oxidative stress and low-grade inflammation, both of which are strongly implicated in lipid abnormalities. Elevated inflammatory cytokines such as tumor necrosis factor (TNF)-α and interleukin (IL)-6 inhibit lipoprotein lipase activity and impair reverse cholesterol transport, contributing to elevated triglycerides and reduced HDL concentrations (68, 69). Epigenetic modifications also play a role. Exposure to arsenic and certain POPs has been associated with altered DNA methylation in genes regulating lipid transport and hepatic metabolism, suggesting a mechanism for persistent metabolic effects even after exposure declines (71).

##### 3.5.2.1 Soil pollutants and hyperlipidemia: evidence from studies

Shao et al. (72) analyzed data from the U.S. National Health and Nutrition Examination Survey (2003–2020) to investigate potential associations between serum lipid parameters and urinary metabolites of organophosphate pesticides, reflecting widespread agricultural use of these compounds. Their findings demonstrated a positive correlation between triglyceride levels and the metabolites diethyl thiophosphate and dimethyl dithiophosphate. In contrast, dimethyl phosphate and diethyl phosphate exhibited non-linear, U-shaped relationships with both total cholesterol and triglyceride concentrations, suggesting complex dose–response dynamics in lipid metabolism associated with pesticide exposure.

In a separate study, Pothu et al. (73) evaluated the relationship between occupational pesticide exposure and serum lipid profiles among 283 agricultural workers in Rajamahendravaram. The investigators observed that individuals with documented pesticide exposure had significantly altered lipid parameters, characterized by elevated total cholesterol levels and reduced HDL concentrations. These findings support the growing body of evidence indicating that chronic exposure to agricultural pesticides may adversely affect lipid metabolism and contribute to cardiometabolic risk in exposed populations.

Yuanyuan et al. (74) investigated the metabolic effects of mixed endocrine-disrupting chemical exposure in a cohort of 144 children, quantifying 36 compounds, including per- and polyfluoroalkyl substances (PFAS), phenols, parabens, PAHs, and phthalates. Their analysis demonstrated positive associations between PFAS concentrations—chemicals frequently detected in animal-derived food residues—and elevated triglyceride levels as well as increased fasting blood glucose, suggesting early-life susceptibility of lipid and glucose metabolism to environmental chemical mixtures.

In an occupational setting, Moon et al. (75) examined 567 individuals exposed to multiple heavy metals—including nickel, mercury, cadmium, lead, iron, and zinc—in the vicinity of the Janghang refinery plant in the Republic of Korea. The cumulative exposure index for these metals was significantly associated with higher total cholesterol concentrations, underscoring the additive cardiometabolic burden of mixed-metal exposure.

Longitudinal data from China by Wang et al. (76) further demonstrated that individuals with persistently elevated BPA levels at baseline and follow-up experienced an additional 6.12% increase in triglycerides and a 2.94% increase in LDL compared with those maintaining lower BPA concentrations. Consistently, Guo et al. (77), analyzing U.S. National Health and Nutrition Examination Survey (2003–2016) data, reported higher urinary BPA levels among participants with hyperlipidemia relative to normolipidemic individuals. Mechanistic work by Moghaddam et al. (78) supports these epidemiologic findings, demonstrating that BPA exposure induced weight gain, increased malondialdehyde levels, and reduced antioxidant defenses—including catalase, superoxide dismutase, glutathione, and total antioxidant capacity—in pancreatic tissue, implicating oxidative stress in metabolic disruption.

Zhou et al. (69) evaluated 1,489 cadmium-exposed workers from smelting facilities in central and southwestern China and reported a mean blood cadmium concentration of 3.61 ± 0.84 µg/L, with a dyslipidemia prevalence of 66.3%. Individuals with dyslipidemia exhibited significantly higher blood cadmium levels. Zhang et al. (79), using National Health and Nutrition Examination Survey data (1999–2018), similarly identified elevated blood lead concentrations—measured by inductively coupled plasma mass spectrometry—in participants with hyperlipidemia.

Arsenic exposure has also been linked to lipid abnormalities. Yue et al. (80), analyzing adolescent data from the National Health and Nutrition Examination Survey (2009–2016), found that creatinine-adjusted urinary arsenic was positively associated with total cholesterol levels. Complementing these findings, Karim et al. (81) reported that arsenic-exposed individuals in Bangladesh exhibited increased oxidized LDL, intercellular adhesion molecule-1, C-reactive protein, and vascular cell adhesion molecule-1 compared with unexposed controls, highlighting arsenic's role in promoting pro-atherogenic and inflammatory lipid alterations.

#### 3.5.3 Soil pollutants and diabetes mellitus/hyperglycemia: pathophysiology and molecular mechanisms

**Figure 4:**
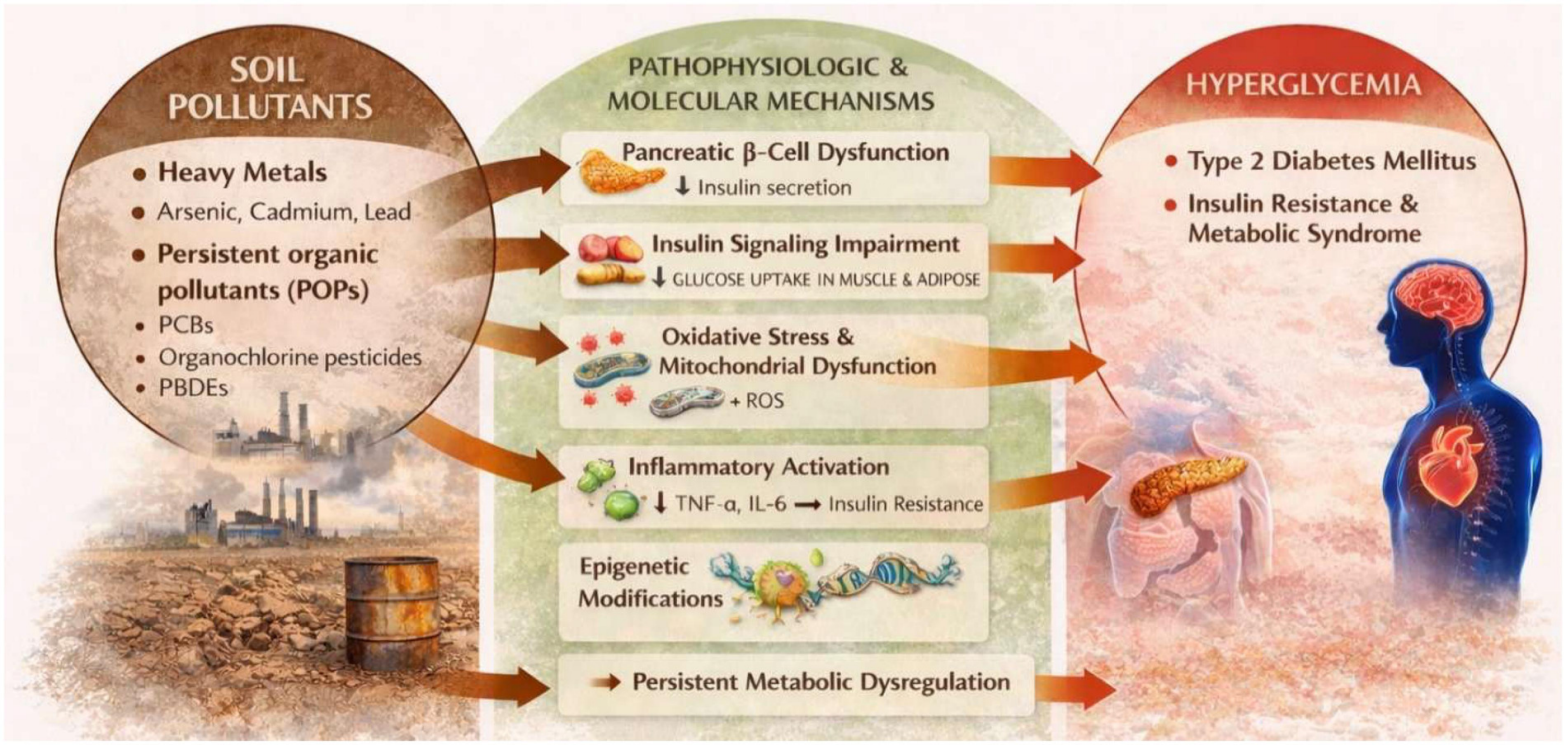
Relationship between soil pollutants and diabetes/hyperglycemia. TNF, tumor necrosis factor-α; IL, interleukin-6; ROS, reactive oxygen species.

The pathophysiologic basis of these associations is multifactorial and involves direct pancreatic, hepatic, and peripheral tissue effects. Arsenic exposure has been shown to impair pancreatic *β*-cell function by inducing oxidative stress, disrupting calcium signaling, and inhibiting insulin gene transcription, thereby reducing insulin secretion (82). Cadmium accumulates in pancreatic islets and hepatocytes, promoting mitochondrial dysfunction, lipid peroxidation, and apoptosis, while also interfering with zinc-dependent enzymatic processes critical for insulin synthesis and storage (83). In peripheral tissues, heavy metals and POPs disrupt insulin signaling pathways by inhibiting phosphorylation of insulin receptor substrate-1 and Akt, thereby impairing glucose uptake in skeletal muscle and adipose tissue (84), as shown in Figure 4.

Several soil pollutants function as endocrine and metabolic disruptors. POPs interact with nuclear receptors, including PPARs and aryl hydrocarbon receptors (AhR), altering transcriptional regulation of genes involved in glucose transport, lipid metabolism, and inflammatory signaling (85). Chronic exposure also promotes low-grade systemic inflammation through activation of NF-*κ*B and increased production of pro-inflammatory cytokines such as TNF-α and IL-6, which are well-recognized mediators of insulin resistance (84).

##### 3.5.3.1 Soil pollutants and diabetes mellitus/hyperglycemia: evidence from studies

Juntarawijit et al. (86) examined the association between agricultural pesticide exposure and diabetes mellitus among residents of Bang Rakam district in Thailand. The study recruited 1,021 non-diabetic controls and 866 patients with diabetes, assessing lifetime exposure to multiple classes of pesticides. Results indicated a positive correlation between diabetes prevalence and exposure to herbicides, insecticides, rodenticides, fungicides, and molluscicides. Similarly, Rebouillat et al. (87) investigated dietary pesticide exposure in relation to type II diabetes risk in European populations. Using non-negative matrix factorization to profile exposures to active chemicals present in European Union-approved pesticides, the study found that individuals with the highest combined exposure to multiple synthetic pesticides had an increased risk of developing type II diabetes mellitus.

In a longitudinal study, Warner et al. (88) followed offspring aged 18 years and older who were prenatally exposed to 2,3,7,8-tetrachlorodibenzo-p-dioxin. A 10-fold increase in maternal dioxin exposure during pregnancy was inversely associated with insulin levels, an effect observed only in female offspring. Experimental studies further support these findings. Tyrrell et al. (89) demonstrated that adult female Zucker diabetic fatty rats exposed to lead via drinking water for 24 weeks developed hyperglycemia within 2 months, followed by glucose intolerance at 3 months. Likewise, Wan et al. (90) reported that Wistar rats exposed to 0.05% lead in drinking water for 28 weeks exhibited elevated fasting plasma glucose, accompanied by increased hepatic glucose production and upregulation of key gluconeogenic enzymes, including glucose-6-phosphatase, fructose-1,6-bisphosphatase, and phosphoenolpyruvate carboxykinase.

### 3.6 Soil pollutants, brain–gut dysbiosis, and neuroinflammation

Growing evidence suggests that environmental soil pollutants contribute to neurological disease risk through disruption of the gut–brain axis, a bidirectional communication network linking intestinal microbiota, immune signaling, and central nervous system (CNS) function. Epidemiologic studies indicate that populations residing in regions with elevated soil contamination by heavy metals and persistent organic pollutants exhibit higher rates of neurodevelopmental delay, cognitive impairment, and neurodegenerative disorders. For example, large cohort analyses in areas with chronic arsenic exposure have demonstrated associations between elevated urinary arsenic and reduced cognitive performance scores in adults and children. Meta-analyses have further linked cadmium and lead exposure to increased risks of cognitive decline and Parkinsonian symptoms (91). In agricultural regions with intensive pesticide use, population-based studies have reported increased incidence of neurobehavioral disorders and higher prevalence of depressive and anxiety symptoms, supporting the role of environmental toxicants in neuropsychiatric outcomes (92).

Heavy metals such as cadmium, arsenic, and mercury disrupt microbial diversity by inhibiting commensal bacterial species and promoting the proliferation of opportunistic or pro-inflammatory taxa. Experimental and human observational studies have demonstrated reductions in beneficial genera such as Lactobacillus and Bifidobacterium in individuals exposed to environmental metals, accompanied by increased intestinal permeability and endotoxemia (93). Persistent organic pollutants, including PCBs and organochlorine pesticides, further alter microbial metabolic pathways involved in SCFA synthesis, thereby impairing epithelial barrier integrity and mucosal immune regulation (94). Reduced SCFA production, particularly butyrate, is strongly associated with systemic inflammation and altered vagal signaling to the brain, key features of gut–brain axis dysregulation.

Neuroinflammation represents a central downstream pathway linking gut dysbiosis to neurological injury. Circulating endotoxins and inflammatory cytokines generated in response to dysbiosis activate microglia and astrocytes, leading to increased production of interleukin-1β, tumor necrosis factor-α, and reactive oxygen species within the CNS (71). Heavy metals can also directly cross the blood–brain barrier, where they induce mitochondrial dysfunction, disrupt calcium signaling, and impair synaptic plasticity. Arsenic and lead exposure have been shown to increase oxidative stress markers and promote protein aggregation pathways implicated in neurodegenerative diseases, including Alzheimer's disease and Parkinson's disease (16). Furthermore, epigenetic alterations—such as DNA methylation changes in genes regulating inflammatory signaling and neuronal differentiation—have been observed in individuals with chronic environmental toxicant exposure, suggesting long-term modulation of neuroimmune pathways (71).

### 3.7 Soil pollutants and stroke: pathophysiology and molecular mechanisms

Heavy metals commonly detected in contaminated soils—including arsenic, cadmium, and lead—exert cumulative toxic effects on the vascular endothelium. Epidemiologic studies from arsenic-endemic regions of Bangladesh and Taiwan have demonstrated that chronic arsenic exposure is associated with a significantly elevated risk of stroke mortality, with hazard ratios ranging from approximately 1.3 to 1.6 in highly exposed populations (95). Similarly, analyses of U.S. population cohorts have reported that higher blood lead levels correlate with increased stroke mortality, with individuals in the highest exposure quartile exhibiting up to a 40% greater risk compared with those in the lowest quartile (96). Meta-analyses examining cadmium exposure have also identified positive associations with ischemic stroke incidence, with pooled relative risks near 1.2–1.3 across observational studies (97).

These associations are supported by well-characterized molecular pathways. Arsenic and cadmium induce endothelial dysfunction by generating reactive oxygen species, reducing nitric oxide bioavailability, and activating pro-inflammatory transcription factors such as NF-*κ*B, which promote vascular inflammation and atherogenesis (51). Lead exposure contributes to hypertension—a major modifiable risk factor for stroke—through disruption of calcium signaling, increased sympathetic activity, and impaired renal sodium handling, all of which elevate systemic vascular resistance (4, 5). Persistent organic pollutants, including PAHs and organochlorine pesticides present in contaminated soils, further exacerbate vascular injury by promoting lipid peroxidation, macrophage activation, and plaque instability, thereby increasing the likelihood of thromboembolic events (50, 63).

At the neurovascular level, several soil pollutants cross or disrupt the blood–brain barrier, amplifying microglial activation and neuroinflammation. Experimental and human biomarker studies demonstrate that chronic exposure to arsenic and PAHs increases circulating inflammatory mediators such as interleukin-6 and C-reactive protein, both independently associated with higher stroke risk and poorer neurological recovery (92, 97, 98). In addition, mitochondrial dysfunction induced by heavy metals impairs neuronal energy metabolism, increasing vulnerability to ischemic injury and worsening poststroke outcomes. Collectively, these converging mechanisms provide biologically plausible evidence that soil pollutant exposure acts as an upstream environmental determinant of cerebrovascular disease, influencing both stroke incidence and clinical prognosis.

## 4 Future perspectives: One Health framework linking soil health, nutrient density, and human and planetary health

The One Health paradigm, which recognizes the interdependence of environmental, animal, and human health, provides a critical framework for understanding how soil health underpins long-term cardiometabolic and neurological outcomes. Healthy soils support balanced microbial ecosystems, efficient nutrient cycling, and improved crop micronutrient composition, thereby strengthening nutrition security and population health. Global assessments indicate that approximately 95% of food production depends directly on soil, yet nearly one-third of the world's soils are moderately to highly degraded due to erosion, contamination, and unsustainable agricultural practices (2, 12). Soil degradation has been linked to measurable declines in essential micronutrients such as zinc, iron, and selenium in staple crops, contributing to “hidden hunger,” which affects more than 2 billion people worldwide and is associated with increased risks of impaired immunity, cardiovascular disease, and neurocognitive dysfunction (99).

Improving soil health has demonstrable benefits for both agricultural productivity and nutritional quality. Regenerative and conservation-based agricultural practices—including crop rotation, organic amendments, and reduced chemical inputs—have been shown to enhance soil organic carbon, microbial diversity, and plant mineral uptake, leading to measurable improvements in nutrient density of food crops and reductions in heavy metal bioavailability (10, 12). These changes have downstream health implications, as diets richer in micronutrients and lower in environmental contaminants are associated with lower prevalence of hypertension, metabolic syndrome, and developmental disorders. Moreover, soil restoration contributes to climate mitigation by increasing carbon sequestration. Estimates suggest that improved soil management could offset up to 5%–10% of global greenhouse gas emissions annually, thereby indirectly reducing climate-related health risks such as heat-related cardiovascular mortality and food insecurity (100).

## 5 Limitations

This narrative review has several limitations. As it did not employ formal systematic review or meta-analysis methodology, the literature search and selection process may be susceptible to selection and publication bias. In addition, the available evidence base is largely observational, with a relative paucity of randomized controlled trials. Moreover, substantial heterogeneity exists across studies in exposure assessment (e.g., soil vs. biomarker measurements), geographic contexts, and analytic methods, limiting comparability and quantitative synthesis. Variability in dose–response characterization, coexposure modeling, and adjustment for socioeconomic and environmental confounders further constrains precision.

## 6 Conclusion

Evidence from environmental, epidemiologic, and clinical research shows that soil functions as a significant determinant of cardiovascular and neurological health. Contaminants such as heavy metals, pesticides, and endocrine-disrupting chemicals enter human systems through food and environmental pathways, contributing to cumulative, low-dose exposures that influence vascular, metabolic, and neuroinflammatory processes across the life course. Soil degradation also reduces nutrient density, compounding risk by weakening antioxidant defenses, impairing endothelial function, and promoting mitochondrial and epigenetic dysfunction. These effects extend beyond overt disease to include subclinical vascular injury, cardiac remodeling, cognitive decline, and increased stroke risk. Recognizing soil quality as a modifiable health determinant and integrating environmental exposure assessment into prevention strategies may improve risk stratification and population health. Protecting soil health, therefore, represents a critical component of long-term cardiovascular and neurological disease prevention. From a public health and policy perspective, integrating soil surveillance, food safety monitoring, and environmental health systems will be essential to operationalize the continuum of healthy soil, healthy food, healthy people, and a healthy planet. Multisectoral collaborations involving agriculture, environmental science, and clinical medicine can facilitate early detection of soil contaminants, strengthen nutrition-sensitive agriculture, and reduce chronic disease burden attributable to environmental exposures. Future research should prioritize longitudinal cohort studies linking soil quality metrics, crop nutrient composition, and clinical outcomes, as well as translational studies exploring microbiome-mediated pathways and epigenetic effects of soil-derived exposures. Such an integrated One Health approach offers a scientifically grounded pathway toward sustainable food systems, improved nutrition security, and reduced global burden of cardiometabolic and neurovascular disease.

## References

[1] Fuller R Landrigan PJ Balakrishnan K Bathan G Bose-O'Reilly S Brauer M Pollution and health: a progress update. Lancet Planet Health. (2022) 6(6):e535–47. 10.1016/S2542-5196(22)00090-0 35594895 PMC11995256

[2] Landrigan PJ Fuller R Acosta NJR Adeyi O Arnold R Basu N The Lancet Commission on pollution and health. Lancet. (2018) 391(10119):462–512. 10.1016/S0140-6736(17)32345-0 29056410

[3] Bhatnagar A. Environmental determinants of cardiovascular disease. Circ Res. (2017) 121(2):162–80. 10.1161/CIRCRESAHA.117.306458 28684622 PMC5777598

[4] Iqubal A Ahmed M Ahmad S Sahoo CR Iqubal MK Haque SE. Environmental neurotoxic pollutants: review. Environ Sci Pollut Res Int. (2020) 27(33):41175–98. 10.1007/s11356-020-10539-z 32820440

[5] Glover F Eisenberg ML Belladelli F Del Giudice F Chen T Mulloy E The association between organophosphate insecticides and blood pressure dysregulation: NHANES 2013–2014. Environ Health. (2022) 21(1):74. 10.1186/s12940-022-00887-3. 35934697 PMC9358881

[6] Lin W Huang Z Zhang W Ren Y. Investigating the neurotoxicity of environmental pollutants using zebrafish as a model organism: a review and recommendations for future work. Neurotoxicology. (2023) 94:235–44. 10.1016/j.neuro.2022.12.009 36581008

[7] Jaishankar M Tseten T Anbalagan N Mathew BB Beeregowda KN. Toxicity, mechanism and health effects of some heavy metals. Interdiscip Toxicol. (2014) 7(2):60–72. 10.2478/intox-2014-0009 26109881 PMC4427717

[8] Aminov Z Haase RF Pavuk M Carpenter DO, Anniston Environmental Health Research Consortium. Analysis of the effects of exposure to polychlorinated biphenyls and chlorinated pesticides on serum lipid levels in residents of Anniston, Alabama. Environ Health. (2013) 12:108. 10.1186/1476-069X-12-108. 24325314 PMC3893492

[9] Arsenescu V Arsenescu RI King V Swanson H Cassis LA. Polychlorinated biphenyl-77 induces adipocyte differentiation and proinflammatory adipokines and promotes obesity and atherosclerosis. Environ Health Perspect. (2008) 116(6):761–8. 10.1289/ehp.10554 18560532 PMC2430232

[10] Iyengar GV Nair PP. Global outlook on nutrition and the environment: meeting the challenges of the next millennium. Sci Total Environ. (2000) 249(1–3):331–46. 10.1016/S0048-9697(99)00529-X 10813462

[11] Zhao FJ Ma Y Zhu YG Tang Z McGrath SP. Soil contamination in China: current status and mitigation strategies. Environ Sci Technol. (2015) 49(2):750–9. 10.1021/es5047099 25514502

[12] Lu Y Song S Wang R Liu Z Meng J Sweetman AJ Impacts of soil and water pollution on food safety and health risks in China. Environ Int. (2015) 77:5–15. 10.1016/j.envint.2014.12.010 25603422

[13] Grifoni M Pellegrino E Arrighetti L Bronco S Pezzarossa B Ercoli L. Interactive impacts of microplastics and arsenic on agricultural soil and plant traits. Sci Total Environ. (2024) 912:169058. 10.1016/j.scitotenv.2023.169058 38070573

[14] Cleaver K Schreiber G. Population, agriculture, and the environment in Africa. Finance Dev. (1992) 29(2):34–5. 12285663

[15] Vogel CFA Van Winkle LS Esser C Haarmann-Stemmann T. The aryl hydrocarbon receptor as a target of environmental stressors—implications for pollution mediated stress and inflammatory responses. Redox Biol. (2020) 34:101530. 10.1016/j.redox.2020.101530 32354640 PMC7327980

[16] Tchounwou PB Yedjou CG Patlolla AK Sutton DJ. Heavy metal toxicity and the environment. Exp Suppl. (2012) 101:133–64. 10.1007/978-3-7643-8340-4_6 22945569 PMC4144270

[17] Chowdhury R Ramond A O'Keeffe LM Shahzad S Kunutsor SK Muka T Environmental toxic metal contaminants and risk of cardiovascular disease: systematic review and meta-analysis. Br Med J. (2018) 362:k3310. 10.1136/bmj.k3310. 30158148 PMC6113772

[18] Nuruzzaman M Bahar MM Naidu R. Diffuse soil pollution from agriculture: impacts and remediation. Sci Total Environ. (2025) 962:178398. 10.1016/j.scitotenv.2025.178398 39808904

[19] Tudi M Daniel Ruan H Wang L Lyu J Sadler R Connell D Agriculture development, pesticide application and its impact on the environment. Int J Environ Res Public Health. (2021) 18(3):1112. 10.3390/ijerph18031112. 33513796 PMC7908628

[20] García MG Sánchez JIL Bravo KAS Cabal MDC Pérez-Santín E. Review: presence, distribution and current pesticides used in Spanish agricultural practices. Sci Total Environ. (2022) 845:157291. 10.1016/j.scitotenv.2022.157291 35835192

[21] Paz-Ferreiro J Gascó G Méndez A Reichman SM. Soil pollution and remediation. Int J Environ Res Public Health. (2018) 15(8):1657. 10.3390/ijerph15081657. 30081583 PMC6121253

[22] Souto AL Sylvestre M Tölke ED Tavares JF Barbosa-Filho JM Cebrián-Torrejón G. Plant-derived pesticides as an alternative to pest management and sustainable agricultural production: prospects, applications and challenges. Molecules. (2021) 26(16):4835. 10.3390/molecules26164835. 34443421 PMC8400533

[23] Ayilara MS Adeleke BS Akinola SA Fayose CA Adeyemi UT Gbadegesin LA Biopesticides as a promising alternative to synthetic pesticides: a case for microbial pesticides, phytopesticides, and nanobiopesticides. Front Microbiol. (2023) 14:1040901. 10.3389/fmicb.2023.1040901. 36876068 PMC9978502

[24] Degrendele C Klánová J Prokeš R Příbylová P Šenk P Šudoma M Current use pesticides in soil and air from two agricultural sites in South Africa: implications for environmental fate and human exposure. Sci Total Environ. (2022) 807(Pt 1):150455. 10.1016/j.scitotenv.2021.150455 34634720

[25] Riedo J Dueñas JF Mbedi S Sparmann S Rillig MC. Abrupt versus gradual application of pesticides: effects on soil bacterial and fungal communities. Environ Pollut. (2025) 383:126859. 10.1016/j.envpol.2025.126859 40684830

[26] Honert C Mauser K Jäger U Brühl CA. Exposure of insects to current use pesticide residues in soil and vegetation along spatial and temporal distribution in agricultural sites. Sci Rep. (2025) 15(1):1817. 10.1038/s41598-024-84811-4. 39838035 PMC11751026

[27] Mesfin YM Mitiku BA Tamrat Admasu H. Veterinary drug residues in food products of animal origin and their public health consequences: a review. Vet Med Sci. (2024) 10(6):e70049. 10.1002/vms3.70049 39334531 PMC11436377

[28] Sin JEV Shen P Teo GS Neo LP Huang L Chua P Surveillance of veterinary drug residues in food commonly consumed in Singapore and assessment of dietary exposure. Heliyon. (2023) 9(11):e21160. 10.1016/j.heliyon.2023.e21160. 37928012 PMC10623269

[29] Martins RR Azevedo VS Pereira AMPT Silva LJG Duarte SC Pena A. Risk assessment of nine coccidiostats in commercial and home raised poultry. J Agric Food Chem. (2021) 69(47):14287–93. 10.1021/acs.jafc.1c05656 34788026

[30] Roila R Branciari R Pecorelli I Cristofani E Carloni C Ranucci D Occurrence and residue concentration of coccidiostats in feed and food of animal origin; human exposure assessment. Foods. (2019) 8(10):477. 10.3390/foods8100477 31614486 PMC6835225

[31] Piątkowska M Gbylik-Sikorska M Gajda A Jedziniak P Błądek T Żmudzki J Multiresidue determination of veterinary medicines in lyophilized egg albumen with subsequent consumer exposure evaluation. Food Chem. (2017) 229:646–52. 10.1016/j.foodchem.2017.02.147 28372226

[32] Moeckel C Breivik K Nøst TH Sankoh A Jones KC Sweetman A. Soil pollution at a major west African E-waste recycling site: contamination pathways and implications for potential mitigation strategies. Environ Int. (2020) 137:105563. 10.1016/j.envint.2020.105563 32106045

[33] Kumar B Verma VK Mishra M Piyush Kakkar V Tiwari A Assessment of persistent organic pollutants in soil and sediments from an urbanized flood plain area. Environ Geochem Health. (2021) 43(9):3375–92. 10.1007/s10653-021-00839-9 33550469

[34] Sun J Pan L Tsang DCW Zhan Y Liu W Wang X Polychlorinated biphenyls in agricultural soils from the Yangtze river Delta of China: regional contamination characteristics, combined ecological effects and human health risks. Chemosphere. (2016) 163:422–8. 10.1016/j.chemosphere.2016.08.038 27565309

[35] Sun J Pan L Zhan Y Lu H Tsang DCW Liu W Contamination of phthalate esters, organochlorine pesticides and polybrominated diphenyl ethers in agricultural soils from the Yangtze River Delta of China. Sci Total Environ. (2016) 544:670–6. 10.1016/j.scitotenv.2015.12.012 26674696

[36] Enjavinejad SM Zahedifar M Moosavi AA Khosravani P. Integrated application of multiple indicators and geographic information system-based approaches for comprehensive assessment of environmental impacts of toxic metals-contaminated agricultural soils and vegetables. Sci Total Environ. (2024) 926:171747. 10.1016/j.scitotenv.2024.171747 38531460

[37] Kapwata T Mathee A Sweijd N Minakawa N Mogotsi M Kunene Z Spatial assessment of heavy metals contamination in household garden soils in rural Limpopo Province, South Africa. Environ Geochem Health. (2020) 42(12):4181–91. 10.1007/s10653-020-00535-0 32062739

[38] Shezi B Street RA Webster C Kunene Z Mathee A. Heavy metal contamination of soil in preschool facilities around industrial operations, Kuils River, Cape Town (South Africa). Int J Environ Res Public Health. (2022) 19(7):4380. 10.3390/ijerph19074380. 35410061 PMC8998666

[39] Yilmaz B Terekeci H Sandal S Kelestimur F. Endocrine disrupting chemicals: exposure, effects on human health, mechanism of action, models for testing and strategies for prevention. Rev Endocr Metab Disord. (2020) 21(1):127–47. 10.1007/s11154-019-09521-z 31792807

[40] Nappi F Barrea L Di Somma C Savanelli M Muscogiuri G Orio F Endocrine aspects of environmental “Obesogen” pollutants. Int J Environ Res Public Health. (2016) 13(8):765. 10.3390/ijerph13080765. 27483295 PMC4997451

[41] Zangiabadian M Jolfayi AG Nejadghaderi SA Amirkhosravi L Sanjari M. The association between heavy metal exposure and obesity: a systematic review and meta-analysis. J Diabetes Metab Disord. (2024) 23(1):11–26. 10.1007/s40200-023-01307-0. 38932800 PMC11196503

[42] Ock J Park CH Choi YH. Environmental exposures to lead, mercury, cadmium, manganese, and arsenic and obesity in Korean adults: Korean National Environmental Health Survey 2009–2017. J Trace Elem Med Biol. (2025) 92:127771. 10.1016/j.jtemb.2025.127771 41106239

[43] Kadawathagedara M de Lauzon-Guillain B Botton J. Environmental contaminants and child’s growth. J Dev Orig Health Dis. (2018) 9(6):632–41. 10.1017/S2040174418000995 30720417

[44] Patra RC Rautray AK Swarup D. Oxidative stress in lead and cadmium toxicity and its amelioration. Vet Med Int. (2011) 2011:1. 10.4061/2011/457327. PMC3087445 21547215

[45] Shan Q Li H Chen N Qu F Guo J. Understanding the multiple effects of PCBs on lipid metabolism. Diabetes Metab Syndr Obes. (2020) 13:3691–702. 10.2147/DMSO.S264851. 33116719 PMC7568599

[46] Skinner MK Guerrero-Bosagna C. Environmental signals and transgenerational epigenetics. Epigenomics. (2009) 1(1):111–7. 10.2217/epi.09.11 20563319 PMC2886501

[47] Islam MR Khan I Attia J Hassan S McEvoy M D'Este C Association between hypertension and chronic arsenic exposure in drinking water: a cross-sectional study in Bangladesh. Int J Environ Res Public Health. (2012) 9(12):4522–36. 10.3390/ijerph9124522. 23222207 PMC3546776

[48] Tellez-Plaza M Navas-Acien A Crainiceanu CM Guallar E. Cadmium exposure and hypertension in the 1999–2004 National Health and Nutrition Examination Survey (NHANES). Environ Health Perspect. (2008) 116(1):51–6. 10.1289/ehp.10764 18197299 PMC2199293

[49] Balali-Mood M Naseri K Tahergorabi Z Khazdair MR Sadeghi M. Toxic mechanisms of five heavy metals: mercury, lead, chromium, cadmium, and arsenic. Front Pharmacol. (2021) 12:643972. 10.3389/fphar.2021.643972. 33927623 PMC8078867

[50] Yang AM Lo K Zheng TZ Yang J Bai Y Feng Y Environmental heavy metals and cardiovascular diseases: status and future direction. Chronic Dis Transl Med. (2020) 6(4):251–9. 10.1016/j.cdtm.2020.02.005. 33336170 PMC7729107

[51] Unsal V Dalkıran T Çiçek M Kölükçü E. The role of natural antioxidants against reactive oxygen Species produced by cadmium toxicity: a review. Adv Pharm Bull. (2020) 10(2):184–202. 10.34172/apb.2020.023 32373487 PMC7191230

[52] Suarez-Lopez JR Amchich F Murillo J Denenberg J. Blood pressure after a heightened pesticide spray period among children living in agricultural communities in Ecuador. Environ Res. (2019) 175:335–42. 10.1016/j.envres.2019.05.030 31150932 PMC6571166

[53] Dong Y Yu Y. Association between non-persistent pesticides and hypertension in adults: insights from NHANES. Int J Environ Health Res. (2025) 35(10):2771–81. 10.1080/09603123.2025.2461108 39900357

[54] Chen H Liang X Chen L Zuo L Chen K Wei Y Associations between household pesticide exposure, smoking and hypertension. Front Public Health. (2022) 10:754643. 10.3389/fpubh.2022.754643. 35273934 PMC8902065

[55] Khalifa HO Shikoray L Mohamed MI Habib I Matsumoto T. Veterinary drug residues in the food chain as an emerging public health threat: sources, analytical methods, health impacts, and preventive measures. Foods. (2024) 13(11):1629. 10.3390/foods13111629. 38890858 PMC11172309

[56] Kuiper HA Noordam MY van Dooren-Flipsen MM Schilt R Roos AH. Illegal use of beta-adrenergic agonists: European community. J Anim Sci. (1998) 76(1):195–207. 10.2527/1998.761195x 9464899

[57] Neary JM Garry FB Gould DH Holt TN Brown RD. The beta-adrenergic agonist zilpaterol hydrochloride may predispose feedlot cattle to cardiac remodeling and dysfunction. F1000Res. (2018) 7:399. 10.12688/f1000research.14313.1

[58] Bae S Hong YC. Exposure to bisphenol A from drinking canned beverages increases blood pressure: randomized crossover trial. Hypertension. (2015) 65(2):313–9. 10.1161/HYPERTENSIONAHA.114.04261 25489056

[59] Bae S Kim JH Lim YH Park HY Hong YC. Associations of bisphenol A exposure with heart rate variability and blood pressure. Hypertension. (2012) 60(3):786–93. 10.1161/HYPERTENSIONAHA.112.197715 22851732

[60] Bae S Lim YH Lee YA Shin CH Oh SY Hong YC. Maternal urinary bisphenol A concentration during midterm pregnancy and children’s blood pressure at age 4. Hypertension. (2017) 69(2):367–74. 10.1161/HYPERTENSIONAHA.116.08281 27920131

[61] Xiong Q Liu X Shen Y Yu P Chen S Hu J Elevated serum bisphenol A level in patients with dilated cardiomyopathy. Int J Environ Res Public Health. (2015) 12(5):5329–37. 10.3390/ijerph120505329. 25996886 PMC4454970

[62] Chen CJ Hsueh YM Lai MS Shyu M-P Chen S-Y Wu M-M Increased prevalence of hypertension and long-term arsenic exposure. Hypertension. (1995) 25(1):53–60. 10.1161/01.HYP.25.1.53 7843753

[63] Ameer SS Engström K Harari F Concha G Vahter M Broberg K. The effects of arsenic exposure on blood pressure and early risk markers of cardiovascular disease: evidence for population differences. Environ Res. (2015) 140:32–6. 10.1016/j.envres.2015.03.010 25825128

[64] Khatun M Haque N Siddique AE Wahed AS Islam MS Khan S Arsenic exposure-related hypertension in Bangladesh and reduced circulating nitric oxide bioavailability. Environ Health Perspect. (2024) 132(4):47003. 10.1289/EHP13018 38573329 PMC10993991

[65] Kaufman JA Mattison C Fretts AM Umans JG Cole SA Voruganti VS Arsenic, blood pressure, and hypertension in the strong heart family study. Environ Res. (2021) 195:110864. 10.1016/j.envres.2021.110864 33581093 PMC8021390

[66] Wang Q Tian H Wang W Liu S Zhang A. The relationship of arsenic exposure with hypertension and wide pulse pressure: preliminary evidence from coal-burning arsenicosis population in southwest China. Toxics. (2023) 11(5):443. 10.3390/toxics11050443. 37235257 PMC10223262

[67] Planchart A Green A Hoyo C Mattingly CJ. Heavy metal exposure and metabolic syndrome: evidence from human and model system studies. Curr Environ Health Rep. (2018) 5(1):110–24. 10.1007/s40572-018-0182-3 29460222 PMC6053628

[68] Navas-Acien A Sharrett AR Silbergeld EK Schwartz BS Nachman KE Burke TA Arsenic exposure and cardiovascular disease: a systematic review of the epidemiologic evidence. Am J Epidemiol. (2005) 162(11):1037–49. 10.1093/aje/kwi330 16269585

[69] Zhou Z Lu YH Pi HF Gao P Li M Zhang L Cadmium exposure is associated with the prevalence of dyslipidemia. Cell Physiol Biochem. (2016) 40(3–4):633–43. 10.1159/000452576 27898410

[70] Lee DH Steffes MW Sjödin A Jones RS Needham LL Jacobs DR Jr. Low dose organochlorine pesticides and polychlorinated biphenyls predict obesity, dyslipidemia, and insulin resistance among people free of diabetes. PLoS One. (2011) 6(1):e15977. 10.1371/journal.pone.0015977. 21298090 PMC3027626

[71] Sobral AF Cunha A Costa I Silva-Carvalho M Silva R Barbosa DJ. Environmental xenobiotics and epigenetic modifications: implications for human health and disease. J Xenobiot. (2025) 15(4):118. 10.3390/jox15040118. 40700165 PMC12286003

[72] Shao W Gong P Zhang K Shen W Liu C Zhao G. Association of organophosphate pesticide exposure with serum lipid profiles in US adults: an analysis of NHANES 2003–2020. Int J Environ Health Res. (2025) 35(10):1–16. 10.1080/09603123.2025.2579082 41163409

[73] Pothu UK Thammisetty AK Nelakuditi LK. Evaluation of cholinesterase and lipid profile levels in chronic pesticide exposed persons. J Family Med Prim Care. (2019) 8(6):2073–8. 10.4103/jfmpc.jfmpc_239_19 31334182 PMC6618179

[74] Zhang Y Zhang B Yang H Liu M Wang J Zhao L Associations of endocrine-disrupting chemicals mixtures with serum lipid and glucose metabolism among overweight/obese and normal-weight children: a panel study. Ecotoxicol Environ Saf. (2025) 294:118077. 10.1016/j.ecoenv.2025.118077 40118019

[75] Moon SI Yim DH Choi K Eom S-Y Choi B-S Park J-D Association between multiple heavy metal exposures and cholesterol levels in residents living near a smelter plant in Korea. J Korean Med Sci. (2024) 39(8):e77. 10.3346/jkms.2024.39.e77. 38442720 PMC10911942

[76] Wang B Wang S Zhao Z Chen Y Xu Y Li M Bisphenol A exposure in relation to altered lipid profile and dyslipidemia among Chinese adults: a repeated measures study. Environ Res. (2020) 184:109382. 10.1016/j.envres.2020.109382 32192991

[77] Guo L Zhao P Xue S Zhu Z. Association of urinary bisphenol A with hyperlipidemia and all-cause mortality: NHANES 2003–2016. PLoS One. (2024) 19(7):e0304516. 10.1371/journal.pone.0304516. 38950289 PMC11216755

[78] Moghaddam HS Samarghandian S Farkhondeh T. Effect of bisphenol A on blood glucose, lipid profile and oxidative stress indices in adult male mice. Toxicol Mech Methods. (2015) 25(7):507–13. 10.3109/15376516.2015.1056395 26376105

[79] Zhang Y Liu W Zhang W Cheng R Tan A Shen S Association between blood lead levels and hyperlipidemiais: results from the NHANES (1999–2018). Front Public Health. (2022) 10:981749. 10.3389/fpubh.2022.981749. 36159291 PMC9500574

[80] Yue Y Nair N Quinones S Kordas K Desai G. Associations of total urinary arsenic with total cholesterol and high-density lipoprotein among 12–17-year-old participants from the 2009–2016 NHANES cycles: a cross-sectional study. Int J Hyg Environ Health. (2022) 242:113950. 10.1016/j.ijheh.2022.113950 35298926

[81] Karim MR Rahman M Islam K Mamun AA Hossain S Hossain E Increases in oxidized low-density lipoprotein and other inflammatory and adhesion molecules with a concomitant decrease in high-density lipoprotein in the individuals exposed to arsenic in Bangladesh. Toxicol Sci. (2013) 135(1):17–25. 10.1093/toxsci/kft130 23761297

[82] Navas-Acien A Silbergeld EK Streeter RA Clark JM Burke TA Guallar E. Arsenic exposure and type 2 diabetes: a systematic review of the experimental and epidemiological evidence. Environ Health Perspect. (2006) 114(5):641–8. 10.1289/ehp.8551 16675414 PMC1459913

[83] Edwards JR Prozialeck WC. Cadmium, diabetes and chronic kidney disease. Toxicol Appl Pharmacol. (2009) 238(3):289–93. 10.1016/j.taap.2009.03.007 19327375 PMC2709710

[84] Ruzzin J. Public health concern behind the exposure to persistent organic pollutants and the risk of metabolic diseases. BMC Public Health. (2012) 12:298. 10.1186/1471-2458-12-298. 22520265 PMC3408385

[85] Nadal A Quesada I Tudurí E Nogueiras R Alonso-Magdalena P. Endocrine-disrupting chemicals and the regulation of energy balance. Nat Rev Endocrinol. (2017) 13(9):536–46. 10.1038/nrendo.2017.51 28524168

[86] Juntarawijit C Juntarawijit Y. Association between diabetes and pesticides: a case-control study among Thai farmers. Environ Health Prev Med. (2018) 23(1):3. 10.1186/s12199-018-0692-5. 29374457 PMC5787249

[87] Rebouillat P Vidal R Cravedi JP Taupier-Letage B Debrauwer L Gamet-Payrastre L Prospective association between dietary pesticide exposure profiles and type 2 diabetes risk in the NutriNet-santé cohort. Environ Health. (2022) 21(1):57. 10.1186/s12940-022-00862-y. 35614475 PMC9131692

[88] Warner M Rauch S Brambilla P Signorini S Mocarelli P Eskenazi B. Prenatal dioxin exposure and glucose metabolism in the Seveso Second Generation study. Environ Int. (2020) 134:105286. 10.1016/j.envint.2019.105286 31726365 PMC6904529

[89] Tyrrell JB Hafida S Stemmer P Adhami A Leff T. Lead (Pb) exposure promotes diabetes in obese rodents. J Trace Elem Med Biol. (2017) 39:221–6. 10.1016/j.jtemb.2016.10.007 27908418

[90] Wan H Wang B Cui Y Wang Y Zhang K Chen C Low-level lead exposure promotes hepatic gluconeogenesis and contributes to the elevation of fasting glucose level. Chemosphere. (2021) 276:130111. 10.1016/j.chemosphere.2021.130111 33691221

[91] Lu L Zhang Y Angley M Bejerano S Brockman JD McClure LA Association of urinary cadmium concentration with cognitive impairment in US adults: a longitudinal cohort study. Neurology. (2024) 103(7):e209808. 10.1212/WNL.0000000000209808 39231381 PMC11373676

[92] Bondy SC. Metal toxicity and neuroinflammation. Curr Opin Toxicol. (2021) 26:8–13. 10.1016/j.cotox.2021.03.008

[93] Giambò F Costa C Teodoro M Fenga C. Role-playing between environmental pollutants and human gut microbiota: a complex bidirectional interaction. Front Med. (2022) 9:810397. 10.3389/fmed.2022.810397. PMC8888443 35252248

[94] Djekkoun N Lalau JD Bach V Depeint F Khorsi-Cauet H. Chronic oral exposure to pesticides and their consequences on metabolic regulation: role of the microbiota. Eur J Nutr. (2021) 60(8):4131–49. 10.1007/s00394-021-02548-6 33837455

[95] Chen Y Graziano JH Parvez F Liu M Slavkovich V Kalra T Arsenic exposure from drinking water and mortality from cardiovascular disease in Bangladesh: prospective cohort study. Br Med J. (2011) 342:d2431. 10.1136/bmj.d2431. 21546419 PMC3088786

[96] Lanphear BP Rauch S Auinger P Allen RW Hornung RW. Low-level lead exposure and mortality in US adults: a population-based cohort study. Lancet Public Health. (2018) 3(4):e177–84. 10.1016/S2468-2667(18)30025-2 29544878

[97] Tellez-Plaza M Jones MR Dominguez-Lucas A Guallar E Navas-Acien A. Cadmium exposure and clinical cardiovascular disease: a systematic review. Curr Atheroscler Rep. (2013) 15(10):356. 10.1007/s11883-013-0356-2 23955722 PMC3858820

[98] Calderón-Garcidueñas L Solt AC Henríquez-Roldán C Torres-Jardón R Nuse B Herritt L Long-term air pollution exposure is associated with neuroinflammation, an altered innate immune response, disruption of the blood-brain barrier, ultrafine particulate deposition, and accumulation of amyloid beta-42 and alpha-synuclein in children and young adults. Toxicol Pathol. (2008) 36(2):289–310. 10.1177/0192623307313011 18349428

[99] Bailey RL West KP Jr Black RE. The epidemiology of global micronutrient deficiencies. Ann Nutr Metab. (2015) 66(Suppl 2):22–33. 10.1159/000371618 26045325

[100] Sá JC Lal R Cerri CC Lorenz K Hungria M de Faccio Carvalho PC. Low-carbon agriculture in South America to mitigate global climate change and advance food security. Environ Int. (2017) 98:102–12. 10.1016/j.envint.2016.10.020 27838119

